# The Use of Upconversion Nanoparticles in Prostate Cancer Photodynamic Therapy

**DOI:** 10.3390/life11040360

**Published:** 2021-04-19

**Authors:** Michał Osuchowski, Filip Osuchowski, Wojciech Latos, Aleksandra Kawczyk-Krupka

**Affiliations:** 1 College of Medical Sciences, University of Rzeszów, 35-310 Rzeszów, Poland; patomorfologia@szpital.rzeszow.pl (M.O.); fosuchowski@ur.edu.pl (F.O.); 2 Specialistic Hospital No. 2, 41-902 Bytom, Poland; wojlatos@gmail.com; 3 Department of Internal Medicine, Angiology and Physical Medicine, Center for Laser Diagnostics and Therapy, Medical University of Silesia in Katowice, 41-902 Bytom, Poland

**Keywords:** upconversion nanoparticles, UCNPs, prostate cancer, photodynamic therapy, PDT

## Abstract

Photodynamic Therapy (PDT) is a cancer treatment that uses light, a photosensitizer, and oxygen to destroy tumors. This article is a review of approaches to the treatment of prostate cancer applying upconversion nanoparticles (UCNPs). UCNPs have become a phenomenon that are rapidly gaining recognition in medicine. They have proven to be highly selective and specific and present a powerful tool in the diagnosis and treatment of prostate cancer. Prostate cancer is a huge health problem in Western countries. Its early detection can significantly improve patients’ prognosis, but currently used diagnostic methods leave much to be desired. Recently developed methodologies regarding UCNP research between the years 2021 and 2014 for prostate cancer PDT will also be discussed. Current limitations in PDT include tissue irradiation with visible wavelengths that have a short tissue penetration depth. PDT with the objectives to synthesize UCNPs composed of a lanthanide core with a coating of adsorbed dye that will generate fluorescence after excitation with near-infrared light to illuminate deep tissue is a subject of intense research in prostate cancer.

## 1. Introduction

Prostate cancer is the second most common malignant neoplasm in men and the fifth cancer-related mortality cause in the world. In Poland and another 83 countries (mainly developed countries), it is the most common malignant solid tumor in men and the number of diagnosed cases is constantly increasing [1,2]. Experts report that the incidence of prostate cancer is constantly increasing. In Poland, this cancer accounts for approx. 20 percent of all diagnosed malignant neoplasms and is currently the most frequently diagnosed malignant neoplasm in men [3]. It seems that reversing the negative trend can be significantly helped by the use of precision medicine, including sensitive early-stage cancer detection, as it has promising potential in terms of lower healthcare costs and better treatment outcomes [4,5,6]. The dynamic development of advanced techniques for the immunolabeling of diseased cells [7], super-resolution imaging [8] and bionanomedicine [9] has laid a good foundation and provided powerful tools for advanced theranostics [10] and the implementation of precision medicine.

## 2. Fluorescence Imaging and Nanoparticles

In cancer cell imaging and theranostics, conventional bio-labeling with bio-reagents including organic dyes, chelates and fluorescent proteins has been used for some time now [11,12]. However, this type of labeling has disadvantages such as photobleaching, chemical and metabolic degradation, and a low signal-to-noise ratio, and therefore, in the case of neoplastic cancers, the use of high-sensitivity testing is severely impeded [13,14]. The use of semiconductor quantum dots (QDs) has partially overcome some of the disadvantages [15] by obtaining high quantum efficiency, bright photoluminescence, good photostability and narrow emission. QDs are therefore widely used in molecular labeling and cellular and in vivo imaging [16]. However, the natural toxicity of QDs, their chemical instability and uncontrolled lifetime [17], as well as the need to stimulate them by UV radiation or short wavelength radiation for the down conversion photon transfer reveal many of the disadvantages of this method, including a low signal-to-noise ratio due to background autofluorescence, depth inherent to the short wavelength of the UV excitation light, and a high probability of cell damage due to prolonged irradiation [18,19,20]. Therefore, there is a need to produce a new class of fluorescent sensors that can identify target cells or tissues with a higher signal-to-noise ratio, stronger light penetration capabilities, better photo stability and negligible tissue photodamage.

## 3. Upconversion Nanoparticles

Upconversion nanoparticles (UCNPs) are nanoscale crystals equipped, for instance, with rare earth ions that have the ability to absorb two or even more low energy photons and therefore are able to emit fluorescence at a shorter wavelength than the excitation wavelength. Normally, UCNPs can absorb in the near infrared (NIR) region and can emit in the visible region of the electromagnetic spectrum [21,22,23]. UCNPs can be characterized by very good chemical and photochemical stability, toxicity and no photobleaching or flicker effects [24,25,26,27]. In addition, in the last fifteen years, thanks to systematic scientific research on UCNPs, it was possible to synthesize monosoluble UCNPs with tunable nanostructure, sizes, shapes, luminescent emitting colors and lifetime [28,29,30,31].

The biological understanding of UCNPs’ interactions with living cells is mostly focused on the functionality of newly developed UCNPs used in biological systems for cancer treatment [32,33]. The delivery of UCNPs to diseased tissue is accomplished systemically by injection, and UPCNs tend to accumulate on or internalize into targeted cells and in healthy tissue to some extent. After delivery to targeted cells, the UCNPs are remotely activated by an external light source producing toxic reactive oxygen species (ROS) in the presence of oxygen. This methodology imparts spatial and temporal selectivity in treatment as the UCNPs localized on targeted cells are activated by selectively illuminating the region of diseased tissue, resulting in necrosis and/or apoptosis. UCNPs are generally hydrophobic due to their capping by long-chain hydrophobic ligands. Engineering the surface of UCNPs to allow their dispersion in aqueous phase for biological applications is required. UCNPs should be nontoxic and biocompatible to cells and to the body. Singlet oxygen is a short-lived ROS (<1 μs in the cell), thus a requirement is to generate ^1^O_2_ within a distance of ca. 20 nm from targets within a cell. There are several probes that can be utilized to detect and quantify the amount of ^1^O_2_ generated by fluorescent UCNPs’ excitation of the photosensitizer in solution and in cells that rely on absorption decay or an increase in probe fluorescence emission. Liu et al. demonstrated the toxic effect of free lanthanide ions on isolated mitochondria [34]. Sabella et al. demonstrated that the toxicity of heavy metal-based NPs was related to their instability at low pH, probably in the lysosomal compartment of the cell [35]. The general mechanism of action of nanoparticles containing heavy metals has also been proposed by Cho et al. [36]. NaGdF4:Yb^3+^,Er^3+^ nanoparticles have shown that gadolinium element exerts cytotoxic activity against isolated mitochondria [37,38]. Cytotoxicity assays measure the loss of cellular or intercellular structures and/or functions. They give an indication of the ability to cause cancer tissue injury, but are not perfect due to the lack of some of the biological protective mechanisms. The toxicity of UCNPs has been investigated with reference to in vitro cytotoxic activity and long-term in vivo toxicity [39,40,41]. The number of prostate cancer cells is low at the early stage, so it is very challenging to detect. In a study by Shi et al. [42], upconversion immune-nanohybrids with sustainable stability in a physiological environment, stable optical properties and highly specific targeting capability for early-stage prostate cancer cell detection were developed. The system was able to specifically detect prostate cancer cells with stable and background-free luminescent signals for highly sensitive prostate cancer cell detection. To facilitate the detection of prostate cancer cells with UINBs, MIL-38-biotinylated antibody was linked to streptavidin coated UCNPs (SA-PEG-UCNPs). The resulting MIL38-biotin-SA-PEG-UCNPs were incubated with prostate cells. This work demonstrates a versatile strategy to develop UCNP-based sustainably stable UINBs for sensitive diseased cell detection [42]. UCNPs can efficiently convert the deeply penetrating near-infrared light to visible wavelengths that can excite the photosensitizer to produce cytotoxic ^1^O_2_, promising their use in the photodynamic therapy (PDT) treatment of pertinent located deep tumors [43]. UCNP-based biosensing and bioassays are emerging as a new thrust in the theranostic field. UCNPs’ attributes such as nonblinking, nonphotobleaching, high chemical stability, sharp emission bands, NIR light excitation, and large anti-stokes emissions make them promising for theranostic applications. UCNPs should be nontoxic and biocompatible to cells and to the body. The monodispersed small size and uniform shape are required to have identical optical properties as well as cellular uptake and biological effects for intracellular theranostics, while a precise stoichiometric composition is necessary to allow control over the concentration of lanthanide dopants to manipulate the optical attributes [32,33,34,35,36,37,38,39,40,41,42,43,44]. The investigation of cytotoxicity is a key factor in determining the potential of employing UCNPs in practical biomedical applications [44]. In particular, the use of UCNPs for treating MB49 cells and 3D spheroids resulted in an IC_50_ of 0.49 and 0.62 mg mL^−1^ [45]. Päkkilä et al. have used UCNPs for the design of heterogeneous microtiter plate immunoassays, e.g., for the detection of prostate-specific antigen (limit of detection, LOD, 0.15 ng mL^−1^/6 pM) [46]. NaLuF4:Gd^3+^/Yb^3+^/Er^3+^ upconversion nanoparticles were prepared by the facile one-step hydrothermal method in aqueous solution. To confirm the cytotoxicity of NaLuF4:Gd^3+^/Yb^3+^/Er^3+^, loaded micelles were prepared and MTT assay was conducted on three different prostate cell lines. The cell viability was improved after the encapsulation of UCNPs in micelles [47]. Other UCNPs were designed based on streptavidin-PEG-neridronate. In a microtiter plate immunoassay, cancer marker prostate-specific antigen (PSA) was detected and counted by wide-field epiluminescence microscopy. The digital detection was 16× more sensitive than the respective analogue readout, thus expanding the limit of detection to the sub-femtomolar concentration range (LOD: 23 fg mL^−1^, 800 µM) [48]. The carboxyl group-functionalized UCNPs were conjugated with anti-PSA detection antibodies and served as the luminescence energy donor, while gold nanoparticles (GNPs) were modified with anti-PSA capture antibodies and acted as the energy acceptor. The range for PSA detection in Tris-buffered saline (TBS) was 0–500 pM and the limit of detection was 1.0 pM, which is much lower than the cutoff level in patients’ serum samples [49]. The use of beta-emitting radionuclide yttrium-90 (90Y) fractionally substituting yttrium in UCNPs resulted in IC_50_ = 0.0024 μg/mL [50].

In UCNP-based detection probes, obtaining a very high sensitivity is possible thanks to the unique anti-stokes shift, which allows the elimination of background noise from the sample under test, and thanks to their long lifetime extends the emission time by several dozen microseconds, allowing time-gated detection to remove autofluorescence and excitation dispersion. This makes UCNPs a promising sensitive nanoprobe for early-stage cancer detection [51].

Phototherapy efficiently generates heat (photothermal therapy, PTT) or reactive oxygen species (ROS) such as singlet oxygen (photodynamic therapy, PDT). Heat damages tissue by protein denaturation and ROS induce DNA damage and impair the DNA repair mechanisms on top of damaging cellular membranes, causing cell damage and apoptosis. UCNPs also deliver drugs such as doxorubicin that disrupt topoisomerase-II-mediated DNA repair and generate ROS.

## 4. UCNPs in Prostate Cancer Imaging

The detection of the desired cells with UCNP probes is possible only when various targeting molecules such as proteins or peptides are coupled to their surface [52,53]. Various strategies for modifying and functionalizing the surface of UCNPs were used to specifically recognize cells and to be able to detect diseases very sensitively. The most promising methods of the chemical modification of UCNPs are: capping ligands removal [54], layer-by-layer assembly [55], optimized salinization chemistry [56], silica coating [57,58], polymer encapsulation [59] and ligand exchange [60,61,62]. Over the last five years, there has been a lot of scientific work describing various methods of producing UCNP-based probes that allow the detection of prostate cancer cells at an early stage of the disease.

One of the well-described methods is upconversion immune-nanohybrids (UINBs) through the one-step ligand exchange strategy. This allows sustainable stability in a body environment, very stable optical properties and specific targeting capability towards prostate cancer cells. In an experiment by Shi et al. [42], streptavidin and an antibody recognizing a prostate cancer antigen (MIL–38) were anchored onto the surface of UCNPs to give UINBs. The system was highly specific in detecting prostate cancer cells with luminescent signals free of background noise and proved to be a highly sensitive prostate cancer detection method [63].

A somewhat comparable method of detecting PSA was presented by Mickert et al. [47] A digital single-molecule upconversion linked immunosorbent assay (ULISA) was used to detect much lower protein concentrations than conventional immunoassays. The authors introduced a UCNP label design based on a streptavidin-PEG-neridronate detection configuration consisting of a biotinylated antibody that decreases nonspecific binding on microtiter plates. The method was used to detect and count by wide-field epiluminescence microscopy the cancer marker prostate-specific antigen (PSA). This digital detection proved to be 16× more sensitive than the respective analogue readout and has a lot of promise in the future [47].

Another group of researchers submitted a work presenting for the first time the applicability of upconversion nanoparticle/graphene oxide sensors for the detection of mRNA-related biomarkers. The PCA3 mRNA biomarker related to prostate cancer was selected to demonstrate the utility of this technique for the early detection of cancer cells. The sensors which are obtained in this way have a couple of important advantages in comparison to previously reported systems. They are highly sensitive with no drawbacks related to biomolecule absorbance interferences. The experiment showed that the sensor platform is highly selective to a selected structure even in complex environments, for example, in blood plasma. These sensors are specific, sensitive, and adaptable for the detection of mRNA biomarkers; thus, they can facilitate the early diagnosis of critical diseases such as prostate cancer [63].

Yu et al. examined the suitability of an upconversion fluorophore, NaYF4:Yb,Er nanoparticles in imaging prostate tumors both in vitro and in vivo. Luminescent signals of CWR22R and LNCaP cell lines of human prostate cancer labeled with NaYF4:Yb,Er nanoparticles were detected with the use of a laser scanning confocal microscope, while Cy3 or FITC was the control probe. After incubation with 30 μg/mL of NaYF4:Yb,Er nanoparticles for 24 h, both CWR22R and LNCaP cells were able to phagocytose the nanoparticles. After excitation with a laser of 980 nm wavelength, the two labeled cell lines exhibited bright green or red fluorescence [64].

In vitro, the CWR22R cancer cell line presented significantly higher uptake of NaYF4:Yb,Er nanoparticles under the same experimental conditions and was chosen for further experiments [64].

Another group of researchers dealing with the use of nanoparticles as a detection court in the detection of prostate cancer has shown that the use of aptamer-based nanoparticles can be a great solution as some types of nucleic acids and relevant nanostructures are already used in the detection and treatment of cancer. Aptamers are single oligonucleotides, with high affinity for the target. They have better stability and storage properties than antibodies. Scientists were able to design a Au-UCNP pyramid model based on various aptamers, which could simultaneously detect two targets with Raman and fluorescence signals, for example, simultaneously detecting PSA and thrombin with high quality results. This method could be useful for the early diagnosis of prostate cancer [65].

Cancer cells incubated with NaYF4:Yb,Er nanoparticles for 24 h in vitro were transplanted to nude mice to simulate a prostate tumor. Fluorescence imaging performed after 4 weeks proved the presence of detectable phagocytosed NaYF4:Yb,Er nanoparticles at the site of the tumor. The result of the study suggests that the described nanoparticles could be successfully used as a marker of neoplastic cells in the assessment of both the primary tumor and distant metastases [48].

A novel idea presented by Li et al. [48] was to combine UCNPs with antibodies for the efficient detection of PSA. They proposed a sandwich-type single-particle enumeration (SPE) immunoassay for the quantitative detection of PSA in a flow chamber. It was possible due to the luminescence resonance energy transfer (LRET) between UCNPs and gold nanoparticles (GNPs). The UCNPs were coupled with PSA detection antibodies (Ab1) and were the luminescence energy donor, while GNPs modified with PSA capture antibodies (Ab2) were acting as the energy acceptor. In the presence of target antigen (PSA), the donor and acceptor were brought close to each other and quenched luminescence could be detected. Thanks to the high selectivity of immunological reaction and upconversion fluorescence from UCNPs, this modality displayed higher specificity towards the targeted antigen molecule in a complex biological surrounding than other detection methods and could be broadly used for quantitative and selective detection of cancer biomarkers [66].

## 5. UCNPs in Prostate Cancer Treatment

One of the experiments paving the way for nanoparticle technology in the treatment of cancer was performed by Guo et al. [66]. The authors noted the limitations of photodynamic therapy due to the limited ability of light to penetrate tissues. Green or blue light is only effective up to 2 mm in depth. Satisfactory tissue penetration could be achieved using near-infrared (NIR) light, and therefore much attention was focused on upconversion nanoparticles that allow the conversion of NIR to visible light, which is most effective in stimulating the photosensitizer to produce reactive oxygen species (ROS). The zinc (II)–phthalocyanine photosensitizer attached to polyethyleneimine-modified sodium yttrium fluoride (NaYF_4_) upconversion nanoparticles proved to be unstable [67].

Cui et al. introduced a multifunctional nanoconstruct composed of upconversion nanoparticles (UCNPs) that transform near-infrared (NIR) light to visible light and a zinc(II) phthalocyanine (ZnPc) photosensitizer. Folate-modified amphiphilic chitosan (FASOC) was placed on UCNPs to make sure that the ZnPc would be hitched on and remain close to the UCNPs to facilitate energy transfer. Confocal microscopy and imaging demonstrated the nanoconstruct’s enhanced tumor selectivity due to the overexpression of folate receptor in cancer cells. ROS generation in deep tissue was higher upon the excitation of UCNPs with the 980 nm light when compared to 660 nm irradiation. NIR light-triggered PDT based on the nanoconstructs showed a tumor inhibition ratio up to 50% compared to 18% with the use of conventional visible light-activated PDT [65].

In 2016, Gao et al. [68] published a paper in which they presented another method of improving the effectiveness of PDT in deep tissue tumors. In this study, a new PDT system c(RGDyK)-SOC-UCNP-ZnPc (R-SUZn) evolved due to the modification of a previous system which consisted of, UCNPs used to convert NIR light into visible light, amphiphilic chitosan for drug delivery, zinc phthalocyanine (ZnPc), and targeting ligand c(RGDyK) aimed at tumor vascular endothelial cells. To simulate deep-seated tumors, 1cm pork tissue was placed on the subcutaneous prostate cancer PC3 line tumors. In addition, a two-step strategy involving PDT and subsequent doxorubicin injection was also tested [68].

R-SUZn administration (20 mg/kg) and irradiation with 660 nm light at an optimal dose of 34 mW/cm^2^ did not inhibit tumor growth. This suggested that the PDT alone failed to induce sufficient cancer cell damage. The next steps were the administration of R-SUZn and doxorubicin without light application, with a light irradiation of 660 nm and NIR light on the PC3 cancer line covered with 1 cm of pork tissue to simulate deep-seated tumors. The most impressive effect (79% tumor inhibition) was achieved in the latter case. The upconversion nanoparticles improved the effectiveness of the photosensitizer stimulated with NIR light in increasing the permeability of blood vessels within the tumor and enhanced the therapeutic effect of doxorubicin. The effect was superior to treatment with doxorubicin alone (56% tumor inhibition) and to 660 nm wavelength light irradiation [68].

Sharipov et al. [44] focused on developing a new method of delivering a drug directly to prostate cancer cells. They introduced phosphate micelles loaded with biocompatible upconversion nanoparticles (UCNPs) and successfully delivered UCNPs to prostate cancer cells via secreted phospholipase A2 (sPLA-2) enzyme cleavage of those micelles. A one-step hydrothermal method was used to prepare carboxyl-functionalized NaLuF_4_:Gd^3+^/Yb^3+^/Er^3+^ upconversion nanoparticles in hydrous solution. For further processing, an optimal reaction time of 12 h was used. This allowed the nanoparticles to present the highest upconversion intensity, along with keeping the smallest possible nanoparticle diameter. To synthesize phosphate surfactant, three steps were used: the synthesis and purification of ethylene glycol stearate, the synthesis and purification of 2-(phosphonooxy)ethyl stearate and the synthesis and purification of PEGylated 2-(phosphonooxy)ethyl stearate. The critical micellar concentration (CMC) and specific activity of sPLA–2 on the surfactant were determined. A sonication of UCNPs with phosphate surfactant at a temperature of 37 °C for 30 min was sufficient to achieve the encapsulation of UCNPs in micelles. Treatment was applied on three cancer lines: HeLa and KB human cervical carcinoma and 22Rv1 human prostate cancer. Bioimaging and cytotoxicity test (MTT assay) confirmed the successful synthesis of a water-soluble, biocompatible phosphate surfactant that can encapsulate and selectively release UCNPs to prostate cancer cells. This delivery system can be used for delivering imaging agents and drugs directly to prostate tumors [44].

Bulmahn et al. [67] concentrated on the problem related to the treatment of advanced or recurrent prostate cancer. In such cases, androgen deprivation therapy (ADT) is usually used first, followed by chemotherapy, if necessary [67]. They introduced a multifunctional hierarchical nanoformulation composed of biodegradable chitosan (CS) coated poly (lactic-co-glycolic acid) (PLGA) nanocarriers loaded with docetaxel (Doc) and interleukin-8 (IL-8) small interfering RNA (siRNA) electrostatically bound to UCNPs that has been developed for the bioimaging and treatment of castration-resistant (recurrent ADT-resistant) prostate cancer (CRPC). This nanoformulation allowed the simultaneous delivery of chemotherapy and gene therapy as well as a bimodal optical and magnetic resonance imaging agent.

PLGA nanocarriers have already been used to deliver hydrophobic drugs, such as DTX, directly to cancer cells, and that is why it was used in this nanoformulation. The addition of a positively charged hydrophilic polymer, which can aid in cellular uptake in the form of chitosan (CS), was necessary due to PLGA’s negative charge and hydrophobic nature.

The nanoformulation was created in multiple steps. First, core nanoparticles were synthesized at room temperature with the use of co-precipitation, followed by Ostwald ripening. Then, the core was coated with a NaYF_4_ shell. Next, Poly-D-Lysine coating was performed to produce a positively charged surface to allow for the electrostatic attachment of IL-8 siRNA. The final result was nanocarriers containing UCNPs, siRNA, DTX or a combination of mentioned particles [67].

One of the drugs that has been used successfully in these patients is docetaxel (DTX). It is highly effective in chemotherapy for prostate lobular adenocarcinoma [69], but the authors suggested that there is still a lot of room for improvement.

For the photodynamic imaging, the sensitizer (Yb^3+^) and activator (Er^3+^) ions were added into the core structure and covered with a NaYF4; Gd 50% shell. The optical properties of these UCNPs were possible due to processes of energy transfer upconversion and excited state absorption that involved Yb^3+^ and Er^3+^ [70]. In order to assess the effectiveness of the nanoformulation, MTT assay was performed and its cytotoxicity was assessed. The benchmark was the concentration needed to achieve 50% of the maximum inhibitory effect IC_50_ compared with that of the unmodified DTX. The encapsulation of UCNP-PDL and the addition of siRNA to the nanoformulation resulted in a ~14,000–fold decrease in this value overall. This nanoformulation combined improved drug delivery with providing bimodal fluorescence imaging and MR contrast enhancement [67].

In recent years, interest in another PDT method has been growing. The possibility of photosensitizer excitation with X rays after converting the applied radiation was tested.

Since the 1960s, research has been carried out on hematoporphyrin derivatives (HpD), and in 2003 Schaffer’s group published an article on the use of porphyrins (including HpD and their active fraction known as Photorfin) as photosensitizers [71]. Other photosensitizers tested for the effectiveness of their X-ray excitation were 5–aminolevulinic acid (5–ALA) as a precursor for a second generation porphyrin family member called protoporphyrin IX, acridine orange (AO), metalloporphyrins, methylene blue (MB) and radachlorin [72].

The results of the tests proved to be promising and were followed by the search for nanoparticles that would facilitate the penetration of the drug into the tissues. The idea was to stimulate a converting substance with X-rays to convert these rays into visible light and irritate the photosensitizer, which then generated ROS that destroyed diseased tissue. Chen and Zhang published this concept in 2006 and described the general idea of PDT associated with the presence of rare earth atoms or heavy metals in an NP with a porphyrin attached to it [70].

For the sake of systematization, the NPs could be divided into four large groups. In many clinical trials, they showed effectiveness in destroying cancer cells and allowed a reduction in the concentration of the photosensitizer used [70].

The NPs that proved effective in damaging prostate cancer cells were Sr_2_MgSi_2_O_7_:Eu^2+^, Dy^3+^ lanthanides NPs with polysiloxane PpIX/FA [72,73] and LaF_3_:Ce^3+^/DMSO/PpIX/PLGA, with an energy of excitation of 90 kV, 5 mA, 0.5 Gy min^−1^ (total of 3 Gy) [74].

According to the authors’ knowledge, no large-scale clinical trials with the use of UCNPs in the diagnosis and treatment of prostate cancer have been conducted so far. The technology is relatively new in the treatment of prostate cancer. It began to develop dynamically only in the last decade. One of the problems is the relatively small number of in vitro experiments carried out so far. Most authors synthesize their own nanoconstruct and then test its effectiveness. Thus far, it has not been possible to reach a consensus on the selection of one conjugate and then test it on a large scale in various research centers in order to assess the reproducibility of the results of the original experiment. Another drawback is the UCNP-based drug concentration in tissues. The cytotoxic effect posed by a high nanoparticle concentration is still very poorly understood and there are no studies that have established a safe dose that can be applied in a living organism.

The effectiveness of treatment also depends on the dose of the conjugate. In vitro experiments have shown to be very promising, but animal testing is imperative to ensure that UCNPs perform as expected in a living organism. An absorption, distribution, metabolism and excretion assessment of the UCNP-based drugs is needed before they can undergo human clinical trials.

Diagnostic tests based on UCNPs for the detection and quantification of prostate cancer markers show greater sensitivity and specificity but are more expensive than those performed on a large scale today. The number of experiments that show the effectiveness of UCNPs in the diagnosis of prostate cancer is still relatively small, but further development of this technique will probably reduce its costs and may introduce it into routine diagnostic procedures over time.

The concept is clinically significant. The effect of ROS produced with PDT will add to the effect of ROS produced by X-rays alone. The use of PDT–X will reduce the dose of radiation during radiotherapy and help avoid some of the side effects of this type of treatment.

## 6. Conclusions

UCNPs represent unique nanomaterials. This review summarizes the advances in the development of UCNPs and emerging applications of prostate cancer treatment. UCNPs’ multifunctional system can be used as a targeted drug delivery system, in which the drug release amount can be monitored by the singlet oxygen generation in cancer cells. In this review article, we consider the progress made in the design and fabrication of upconverting nanoparticles. We believe that this heterogeneous nanomaterial has the potential to create a new level of prostate cancer drug delivery.

## References

[1] McGuire S. (2016). World Cancer Report 2014. Geneva, Switzerland: World Health Organization, International Agency for Research on Cancer, WHO Press, 2015. Adv. Nutr..

[2] Bray F., Ferlay J., Soerjomataram I., Siegel R.L., Torre L.A., Jemal A. (2018). Global cancer statistics 2018: GLOBOCAN estimates of incidence and mortality worldwide for 36 cancers in 185 countries. CA Cancer J. Clin..

[3] Czaderny K. (2018). High prostate cancer mortality in Poland. A spatial, temporal and structural analysis. Prz. Epidemiol..

[4] Collins F.S., Varmus H. (2015). A New Initiative on Precision Medicine. N. Engl. J. Med..

[5] König I.R., Fuchs O., Hansen G., Von Mutius E., Kopp M.V. (2017). What is precision medicine?. Eur. Respir. J..

[6] Jung B., Zhu Y., Santiago J.G. (2007). Detection of 100 aM Fluorophores Using a High-Sensitivity On-Chip CE System and Transient Isotachophoresis. Anal. Chem..

[7] Yang Y., Zhao M., Liu X., Ge P., Zheng F., Chen T., Sun X. (2018). Two-way detection of image features and immunolabeling of lymphoma cells with one-step microarray analysis. Biomicrofluidics.

[8] Piliarik M., Sandoghdar V. (2014). Direct optical sensing of single unlabelled proteins and super-resolution imaging of their binding sites. Nat. Commun..

[9] Liu Y., Xu C.-F., Iqbal S., Yang X.-Z., Wang J. (2017). Responsive Nanocarriers as an Emerging Platform for Cascaded Delivery of Nucleic Acids to Cancer. Adv. Drug Deliv. Rev..

[10] Kulkarni H.R., Singh A., Langbein T., Schuchardt C., Mueller D., Zhang J., Lehmann C., Baum R.P. (2018). Theranostics of prostate cancer: From molecular imaging to precision molecular radiotherapy targeting the prostate specific membrane antigen. Br. J. Radiol..

[11] Lei Q., Zhao L., Ye S., Sun Y., Xie F., Zhang H., Zhou F., Wu S. (2019). Rapid and quantitative detection of urinary Cyfra21-1 using fluorescent nanosphere-based immunochromatographic test strip for diagnosis and prognostic monitoring of bladder cancer. Artif. Cells Nanomed. Biotechnol..

[12] Mallia R.J., McVeigh P.Z., Fisher C.J., Veilleux I., Wilson B.C. (2015). Wide-field multiplexed imaging of EGFR-targeted cancers using topical application of NIR SERS nanoprobes. Nanomedicine.

[13] Hama Y., Urano Y., Koyama Y., Bernardo M., Choyke A.P.L., Kobayashi H. (2006). A Comparison of the Emission Efficiency of Four Common Green Fluorescence Dyes after Internalization into Cancer Cells. Bioconj. Chem..

[14] Ankersmit M., Bonjer H.J., Hannink G., Schoonmade L.J., Van Der Pas M.H.G.M., Meijerink W.J.H.J. (2019). Near-infrared fluorescence imaging for sentinel lymph node identification in colon cancer: A prospective single-center study and systematic review with meta-analysis. Tech. Coloproctol..

[15] Li J., Wu D., Miao Z., Zhang Y. (2010). Preparation of quantum dot bioconjugates and their applications in bio-imaging. Curr. Pharm. Biotechnol..

[16] Hoshyar N., Gray S., Han H., Bao G. (2016). The effect of nanoparticle size on in vivo pharmacokinetics and cellular interaction. Nanomedicine.

[17] Hardman R. (2006). A Toxicologic Review of Quantum Dots: Toxicity Depends on Physicochemical and Environmental Factors. Environ. Health Perspect..

[18] Michalet X., Pinaud F.F., Bentolila L.A., Tsay J.M., Doose S., Li J.J., Sundaresan G., Wu A.M., Gambhir S.S., Weiss S. (2005). Quantum Dots for Live Cells, in Vivo Imaging, and Diagnostics. Science.

[19] Jaiswal J.K., Simon S.M. (2015). Imaging Live Cells Using Quantum Dots. Cold Spring Harb. Protoc..

[20] Choi A.O., Neibert K.D., Maysinger D. (2014). Quantum Dots for Imaging Neural Cells In Vitro and In Vivo. Adv. Struct. Saf. Stud..

[21] Zhou B., Shi B., Jin D., Liu X. (2015). Controlling upconversion nanocrystals for emerging applications. Nat. Nanotechnol..

[22] Li H., Wang X., Huang D., Chen G. (2020). Recent advances of lanthanide-doped upconversion nanoparticles for biological ap-plications. Nanotechnology.

[23] Qin X., Xu J., Wu Y., Liu X. (2019). Energy-Transfer Editing in Lanthanide-Activated Upconversion Nanocrystals: A Toolbox for Emerging Applications. ACS Cent. Sci..

[24] Qiu X., Zhu X., Su X., Xu M., Yuan W., Liu Q., Xue M., Liu Y., Feng W., Li F. (2019). Near-Infrared Upconversion Luminescence and Bioimaging In Vivo Based on Quantum Dots. Adv. Sci..

[25] Wang Y.-F., Liu G.-Y., Sun L.-D., Xiao J.-W., Zhou J.-C., Yan C.-H. (2013). Nd^3+^ -Sensitized Upconversion Nanophosphors: Efficient In Vivo Bioimaging Probes with Minimized Heating Effect. ACS Nano.

[26] Zhao F., Li Z., Wang L., Hu C., Zhang Z., Li C., Qu L. (2015). Supramolecular quantum dots as biodegradable nano-probes for upconversion-enabled bioimaging. Chem. Commun..

[27] Liu J., Liu Y., Bu W., Bu J., Sun Y., Du J., Shi J. (2014). Ultrasensitive Nanosensors Based on Upconversion Nanoparticles for Selective Hypoxia Imaging in Vivo upon Near-Infrared Excitation. J. Am. Chem. Soc..

[28] Xia A., Chen M., Gao Y., Wu D., Feng W., Li F. (2012). Gd^3+^ complex-modified NaLuF4-based upconversion nanophosphors for trimodality imaging of NIR-to-NIR upconversion luminescence, X-Ray computed tomography and magnetic resonance. Biomaterials.

[29] Kong M., Gu Y., Liu Y., Shi Y., Wu N., Feng W., Li F. (2019). Luminescence Lifetime–Based In Vivo Detection with Responsive Rare Earth–Dye Nanocomposite. Small.

[30] Kataoka T., Abe S., Tagaya M. (2019). Surface-Engineered Design of Efficient Luminescent Europium(III) Complex-Based Hydroxyapatite Nanocrystals for Rapid HeLa Cancer Cell Imaging. ACS Appl. Mater. Interfaces.

[31] Rafique R., Baek S.H., Phan L.M.T., Chang S.-J., Gul A.R., Park T.J. (2019). A facile hydrothermal synthesis of highly luminescent NaYF4:Yb^3+^/Er^3+^ upconversion nanoparticles and their biomonitoring capability. Mater. Sci. Eng. C.

[32] Gnach A., Lipinski T., Bednarkiewicz A., Rybka J., Capobianco J.A. (2015). Upconverting nanoparticles: Assessing the toxicity. Chem. Soc. Rev..

[33] Sun Y., Feng W., Yang P., Huang C., Li F. (2014). The biosafety of lanthanide upconversion nanomaterials. Chem. Soc. Rev..

[34] Liu H., Yuan L., Yang X., Wang K. (2003). La^3+^, Gd^3+^ and Yb^3+^ induced changes in mitochondrial structure, membrane permeability, cytochrome c release and intracellular ROS level. Chem. Biol. Interact..

[35] Sabella S., Carney R.P., Brunetti V., Malvindi M.A., Al-Juffali N., Vecchio G., Janes S.M., Bakr O.M., Cingolani R., Stellacci F. (2014). A geeral mechanism for intracellular toxicity of metal-containing nanoparticles. Nanoscale.

[36] Cho W.-S., Duffin R., Howie S.E., Scotton C.J., Wallace W.A., MacNee W., Bradley M., Megson I.L., Donaldson K. (2011). Progressive severe lung injury by zinc oxide nanoparticles; the role of Zn^2+^ dissolution inside lysosomes. Part. Fibre Toxicol..

[37] Morrison D.E., Aitken J.B., de Jonge M.D., Ioppolo J.A., Harris H.H., Rendina L.M. (2014). High mitochondrial accumulation of new gadolinium(III) agents within tumour cells. Chem. Commun..

[38] Zhao J., Zhou Z.-Q., Jin J.-C., Yuan L., He H., Jiang F.-L., Yang X.-G., Dai J., Liu Y. (2014). Mitochondrial dysfunction induced by different concentrations of gadolinium ion. Chemosphere.

[39] Xiong L., Yang T., Yang Y., Xu C., Li F. (2010). Long-term in vivo biodistribution imaging and toxicity of polyacrylic acid-coated upconversion nanophosphors. Biomaterials.

[40] Gai S.L., Yang P.P., Li C.X., Wang W.X., Dai Y.L., Niu N., Lin J. (2010). Synthesis of Magnetic, Up-Conversion Luminescent, and Mesoporous Core–Shell-Structured Nanocomposites as Drug Carriers. J. Adv. Funct. Mater..

[41] Ukonaho T., Rantanen T., Jämsen L., Kuningas K., Päkkilä H., Lövgren T., Soukka T. (2007). Comparison of infrared-excited up-converting phosphors and europium nanoparticles as labels in a two-site immunoassay. Anal. Chim. Acta.

[42] Shi Y., Shi B., Dass A.V.E., Lu Y., Sayyadi N., Kautto L., Willows R.D., Chung R., Piper J., Nevalainen H. (2016). Stable Upconversion Nanohybrid Particles for Specific Prostate Cancer Cell Immunodetection. Sci. Rep..

[43] Zhang P., Steelant W., Kumar M., Scholfield M. (2007). Versatile Photosensitizers for Photodynamic Therapy at Infrared Excitation. J. Am. Chem. Soc..

[44] Sharipov M., Tawfik S.M., Gerelkhuu Z., Huy B.T., Lee Y.-I. (2017). Phospholipase A2-Responsive Phosphate Micelle-Loaded UCNPs for Bioimaging of Prostate Cancer Cells. Sci. Rep..

[45] Zhang Z., Rahmat J.N., Mahendran R., Zhang Y. (2020). Controllable Assembly of Upconversion Nanoparticles Enhanced Tumor Cell Penetration and Killing Efficiency. Adv. Sci..

[46] Päkkilä H., Ylihärsilä M., Lahtinen S., Hattara L., Salminen N., Arppe R., Lastusaari M., Saviranta P., Soukka T. (2012). Quantitative multianalyte microarray immunoassay utilizing upconverting phosphor technology. Anal. Chem..

[47] Mickert M.J., Farka Z., Kostiv U., Hlaváček A., Horák D., Skládal P., Gorris H.H. (2019). Measurement of Sub-femtomolar Con-centrations of Prostate-Specific Antigen through Single-Molecule Counting with an Upconversion-Linked Immunosorbent Assay. Anal. Chem..

[48] Li X., Wei L., Pan L., Yi Z., Wang X., Ye Z., Xiao L., Li H.-W., Wang J. (2018). Homogeneous Immunosorbent Assay Based on Single-Particle Enumeration Using Upconversion Nanoparticles for the Sensitive Detection of Cancer Biomarkers. Anal. Chem..

[49] Farka Z., Mickert M.J., Hlaváček A., Skládal P., Gorris H.H. (2017). Single Molecule Upconversion-Linked Immunosorbent Assay with Extended Dynamic Range for the Sensitive Detection of Diagnostic Biomarkers. Anal. Chem..

[50] Guryev E.L., Volodina N.O., Shilyagina N.Y., Gudkov S.V., Balalaeva I.V., Volovetskiy A.B., Lyubeshkin A.V., Sen A.V., Ermilov S.A., Vodeneev V.A. (2018). Radioactive (90/Y) upconversion na-noparticles conjugated with recombinant targeted toxin for synergistic nanotheranostics of cancer. Proc. Natl. Acad. Sci. USA.

[51] Lu Y., Lu J., Zhao J., Cusido J., Raymo F.M., Yuan J., Yang S., Leif R.C., Huo Y., Piper J.A. (2014). On-the-fly decoding luminescence lifetimes in the microsecond region for lanthanide-encoded suspension arrays. Nat. Commun..

[52] Qu Q., Si Y., Xuan H., Zhang K., Chen X., Ding Y., Feng S., Yu H.-Q., Abdullah M.A., Alamry K.A. (2018). Dendritic core-shell silica spheres with large pore size for separation of biomolecules. J. Chromatogr. A.

[53] Lu J., Chen Y., Liu D., Ren W., Lu Y., Shi Y., Piper J., Paulsen I., Jin D. (2015). One-Step Protein Conjugation to Upconversion Nanoparticles. Anal. Chem..

[54] Nyk M., Kumar R., Ohulchanskyy T.Y., Bergey E.J., Prasad P.N. (2008). High Contrast in Vitro and in Vivo Photoluminescence Bioimaging Using Near Infrared to Near Infrared Up-Conversion in Tm^3+^ and Yb^3+^ Doped Fluoride Nanophosphors. Nano Lett..

[55] Coustet M., Irigoyen J., García T.A., Murray R.A., Romero G., Cortizo M.S., Knoll W., Azzaroni O., Moya S.E. (2014). Layer-by-layer assembly of polymersomes and polyelectrolytes on planar surfaces and microsized colloidal particles. J. Colloid Interface Sci..

[56] Rantanen T., Järvenpää M.-L., Vuojola J., Arppe R., Kuningas K., Soukka T. (2009). Upconverting phosphors in a dual-parameter LRET-based hybridization assay. Analyst.

[57] Li Z., Zhang Y. (2006). Monodisperse Silica-Coated Polyvinylpyrrolidone/NaYF4 Nanocrystals with Multicolor Upconversion Fluorescence Emission. Angew. Chem. Int. Ed..

[58] Fathy M.M., Fahmy H.M., Saad O.A., Elshemey W.M. (2019). Silica-coated iron oxide nanoparticles as a novel nano-radiosensitizer for electron therapy. Life Sci..

[59] Jiang G., Pichaandi J., Johnson N.J.J., Burke R.D., Van Veggel F.C.J.M. (2012). An Effective Polymer Cross-Linking Strategy to Obtain Stable Dispersions of Upconverting NaYF4 Nanoparticles in Buffers and Biological Growth Media for Biolabeling Applications. Langmuir.

[60] Kong W., Sun T., Chen B., Chen X., Ai F., Zhu X., Li M., Zhang W., Zhu G., Wang F. (2016). A General Strategy for Ligand Exchange on Upconversion Nanoparticles. Inorg. Chem..

[61] Chen Y., D’Amario C., Gee A., Duong H.T., Shimoni O., Valenzuela S.M. (2020). Dispersion stability and biocompatibility of four ligand-exchanged NaYF4: Yb, Er upconversion nanoparticles. Acta Biomater..

[62] Dong A., Ye X., Chen J., Kang Y., Gordon T., Kikkawa J.M., Murray C.B. (2011). A Generalized Ligand-Exchange Strategy Enabling Sequential Surface Functionalization of Colloidal Nanocrystals. J. Am. Chem. Soc..

[63] Hao T., Wu X., Xu L., Liu L., Ma W., Kuang H., Xu C. (2017). Ultrasensitive Detection of Prostate-Specific Antigen and Thrombin Based on Gold-Upconversion Nanoparticle Assembled Pyramids. Small.

[64] Yu Y., Huang T., Wu Y., Ma X., Yu G., Qi J. (2013). In-Vitro and In-Vivo Imaging of Prostate Tumor Using NaYF4: Yb, Er Up-Converting Nanoparticles. Pathol. Oncol. Res..

[65] Cui S., Yin D., Chen Y., Di Y., Chen H., Ma Y., Achilefu S., Gu Y. (2013). In Vivo Targeted Deep-Tissue Photodynamic Therapy Based on Near-Infrared Light Triggered Upconversion Nanoconstruct. ACS Nano.

[66] Guo H., Qian H., Idris N.M., Zhang Y. (2010). Singlet oxygen-induced apoptosis of cancer cells using upconversion fluorescent nanoparticles as a carrier of photosensitizer. Nanomed. Nanotechnol. Biol. Med..

[67] Bulmahn J.C., Kutscher H.L., Cwiklinski K., Schwartz S.A., Prasad P.N., Aalinkeel R. (2020). A Multimodal Theranostic Nanoformulation that Dramatically Enhances Docetaxel Efficacy Against Castration Resistant Prostate Cancer. J. Pharm. Sci..

[68] Gao W., Wang Z., Lv L., Yin D., Chen D., Han Z., Ma Y., Zhang M., Yang M., Gu Y. (2016). Photodynamic Therapy Induced Enhancement of Tumor Vasculature Permeability Using an Upconversion Nanoconstruct for Improved Intratumoral Nanoparticle Delivery in Deep Tissues. Theranostics.

[69] Larue L., Ben Mihoub A., Youssef Z., Colombeau L., Acherar S., André J.-C., Arnoux P., Baros F., Vermandel M., Frochot C. (2018). Using X-rays in photodynamic therapy: An overview. Photochem. Photobiol. Sci..

[70] Chen W., Zhang J. (2006). Using Nanoparticles to Enable Simultaneous Radiation and Photodynamic Therapies for Cancer Treatment. J. Nanosci. Nanotechnol..

[71] Schaffer M., Ertl-Wagner B., Kulka U., Hofstetter A., Duhmke E., Jori G. (2003). Porphyrins as Radiosensitizing Agents for Solid Neoplasms. Curr. Pharm. Des..

[72] Zou X., Yao M., Ma L., Hossu M., Han X., Juzenas P., Chen W. (2014). X-ray-induced nanoparticle-based photodynamic therapy of cancer. Nanomedicine.

[73] Cornford P., Bellmunt J., Bolla M., Briers E., De Santis M., Gross T., Henry A.M., Joniau S., Lam T.B., Mason M.D. (2017). EAU-ESTRO-SIOG Guidelines on Prostate Cancer. Part II: Treatment of Relapsing, Metastatic, and Castration-Resistant Prostate Cancer. Eur. Urol..

[74] Homayoni H., Ma L., Zhang J., Sahi S.K., Rashidi L.H., Bui B., Chen W. (2016). Synthesis and conjugation of Sr_2_MgSi_2_O_7_:Eu^2+^, Dy^3+^ water soluble afterglow nanoparticles for photodynamic activation. Photodiagn. Photodyn. Ther..

